# A noninvasive bioengineering technology for testing medical monitoring capabilities for conditions of human hypovolemia and hypotension

**DOI:** 10.3389/fbioe.2026.1761484

**Published:** 2026-03-13

**Authors:** Victor A. Convertino, Eric J. Snider

**Affiliations:** 1 Battlefield Health and Trauma Center for Human Integrative Physiology, US Army Institute of Surgical Research, San Antonio, TX, United States; 2 Department of Medicine, Uniformed Services University of the Health Sciences, Bethesda, MD, United States; 3 Department of Emergency Medicine, University of Texas Health, San Antonio, TX, United States; 4 Organ Support and Automation Technology Research Department, San Antonio, TX, United States; 5 Department of Surgery, University of Texas Health, San Antonio, TX, United States

**Keywords:** hemorrhage, hypotension, hypovolemia, lower body negative pressure, medical monitoring, shock, wearable sensors

## Abstract

Testing new medical monitors and wearable sensors designed to assess patient status under conditions of hypovolemia and/or hypotension are necessary to improve clinical outcomes of individuals with hemorrhagic injuries. Lower body negative pressure (LBNP) has emerged as a bioengineering tool that can induce progressive reductions in central blood volume similar to those experienced by patients during the early stages of physiological compensation during blood loss. The objective of this review is to develop a working framework for biomedical engineering research involving a safe noninvasive human hypovolemia model for the systematic testing of medical monitoring sensors and devices. As a testing tool, this paper provides a summary of the safety and advantages of using LBNP to avoid the use of blood withdrawal approaches compared to actual controlled hemorrhage. In this regard, LBNP provides a safe and non-invasive technology for testing advanced medical monitoring technologies with the potential to improve emergency clinical outcomes.

## 1 Introduction

We previously published a paper that provided a systematic technical evaluation of lower body negative pressure (LBNP) as a human model of hemorrhage commonly accepted based on the FDA-recognized “Standard for Assessing Credibility of Modeling through Verification and Validation for Medical Devices” (ASME standard VandV 40) (Convertino et al., 2023). We chose to focus this review paper on the description of LBNP with its advantages and disadvantages that can be used to test new emerging machine learning algorithms and wearable technologies for advanced medical monitoring.

Historically, the development of accurate decision support monitoring systems has proven to be inadequate because of the use of animal models that have failed to translate to humans (Convertino et al., 2021). LBNP represents a physiological model with the capability to induce experimentally controlled central hypovolemia (i.e., reduced central blood volume) (Convertino et al., 2023; Convertino et al., 2021; Goswami et al., 2019; Cooke et al., 2004; Convertino et al., 2016; Schiller et al., 2017; Convertino et al., 2013) that can lead to symptoms associated with progression to the onset of de-compensated shock (Cooke et al., 2004). At least 20 articles have been published that provide data to support the notion that LBNP can be used as an experimental model for the study of human hemorrhage (Convertino et al., 2021; Goswami et al., 2019). The goal of this review is to provide a systematic technical description of LBNP that can be used by the engineering community for testing new monitoring technologies. We highlight the noninvasive nature of LBNP as a testing platform for the safe study of physiological conditions of hypovolemia and hemorrhage in humans.

## 2 Description of the LBNP technology

**FIGURE 1:**
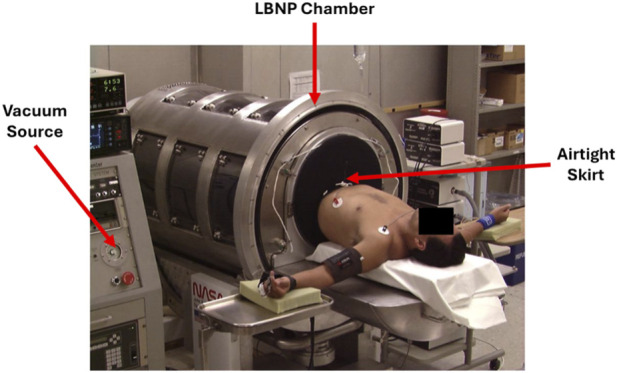
Diagram of Lower Body Negative Pressure Chamber with vacuum source and airtight skirt placed around the human participant shown.

The acronym LBNP accurately describes the function of the technology in that the lower body below the waist is placed in an airtight chamber that is sealed with the use of a specially designed skirt around the torso of the body below the waist (Figure 1). The chamber is connected to a vacuum source that allows for the creation of negative pressure within the chamber. The negative pressure serves to redistribute blood from the upper part of the body that is outside the chamber to the lower portion of the body inside the chamber. The redistribution of blood volume from the upper to the lower portion of the body results in central hypovolemia (lower central blood volume) which is reversible by immediate termination of the negative chamber pressure. As such, LBNP provides a capability to safely test diagnostic devices and wearable sensors during simulated hemorrhage. For instance, the United States Army Medical Capability Directorate has introduced the development of a noninvasive wearable Medical Trauma Sensor (MTS) for early detection of central hypovolemia and prediction of hemorrhagic shock that can be used to inform and improve patient outcomes. LBNP could provide a testing platform for assessments of medical requirements such as the MTS to inform military and civilian medical personnel of the need for earlier lifesaving interventions as well as a capability of evaluating head-to-head performance of new monitoring and detection devices in a fashion that depicts progressive hypovolemia.

The progressive hypovolemia condition allows for targeting the severity of simulated blood loss with stepwise increases in the vacuum pressure, which in turn results in more pronounced central hypovolemia following equilibration at each pressure step. This unique feature of LBNP allows for determining the clinical tolerance for each individual, indicating the body’s physiological failure to sustain adequate blood flow and oxygen delivery to the brain, resulting in subsequent hemodynamic instability and decompensation. As such, LBNP can be used to provide a controlled presence and progression of reduced central blood volume in humans with utility for use in testing any monitoring technologies or sensors.

## 3 Foundational principles of LBNP

Although LBNP has been used to investigate the physiology of central hypovolemia that is fundamental to the understanding of hemorrhage, there are limited studies that have been conducted with direct comparisons between hemorrhage and LBNP (Convertino et al., 2021). However, there are two key investigations involving the direct comparison of physiological responses in which non-human primates (NHP) and healthy human subjects served as their own controls by undergoing exposures to both LBNP and hemorrhage (Johnson et al., 2014; Hinojosa-Laborde et al., 2014). The results from these two investigations provide the most compelling data to support the use of LBNP as a surrogate for the study of hemorrhage physiology.

- Can be used to create a clinical outcome of decompensated shock during a hemorrhagic or hypovolemic event.
- Can replace an evaluation that must typically be conducted in human studies that relies on the high-risk experimental condition involved with blood removal.
- Could reduce or minimize the number of animals which must be used in studies of hemorrhage conducted in support of device approvals or clearances.
- Can be used to assess and/or support demonstration of medical technology safety, effectiveness, or device performance while providing support to regulatory decision making.

## 4 Qualitative overview of LBNP for simulation of hemorrhage

Physiological responses created by exposure to LBNP have been extensively studied (Convertino et al., 2023; Convertino et al., 2021; Goswami et al., 2019; Cooke et al., 2004; Convertino et al., 2016; Schiller et al., 2017; Convertino et al., 2013). Although there has been interest in demonstrating that LBNP-induced central hypovolemia may replicate clinical manifestations during the early compensated phase of blood loss and onset of decompensated hemorrhagic shock in humans, there are a limited number of published data in which physiological responses are directly compared between progressive levels of LBNP and controlled blood withdrawal, particularly in humans. Using comparisons of 27 physiological responses, Table 1 provides a qualitative assessment of hypovolemia created by LBNP compared with hypovolemia created through controlled hemorrhage in healthy human subjects and non-human primates.

**TABLE 1:** Qualitative comparisons in cardiorespiratory, hematological, metabolic, neuroendocrine, and compensatory reserve responses to hemorrhage and lower body negative pressure (LBNP).

Parameter	HEM equivalent	Δ with HEM	Δ with LBNP	References 1	HT vs. LT distinction	References 2
ABP	Yes	↓	↓	Cooke et al. (2004)* Johnson et al. (2014)* Hinojosa-Laborde et al. (2014) van Helmond et al. (2015)* van Helmond et al. (2016)*	No	Convertino et al. (2021)* Schiller et al. (2017)* Convertino et al. (2013)* Xiang et al. (2018)* Schiller et al. (2017)*
CVP	Yes	↓	↓	Cooke et al. (2004)* Johnson et al. (2014)* Hinojosa-Laborde et al. (2014) van Helmond et al. (2015)* van Helmond et al. (2016)* Rea et al. (1991)*	Unk	-
PP	Yes	↓	↓	Johnson et al. (2014)* Hinojosa-Laborde et al. (2014)	No	Convertino et al. (2013)*
CO	Yes	↓	↓	Johnson et al. (2014)* Hinojosa-Laborde et al. (2014) van Helmond et al. (2015)* van Helmond et al. (2016)*	No	Schiller et al. (2017) Koons et al. (2020) Convertino and Sather (2000)*
SV	Yes	↓	↓	Johnson et al. (2014)* Hinojosa-Laborde et al. (2014) Xiang et al. (2018)* van Helmond et al. (2015)* van Helmond et al. (2016)*	No	Koons et al. (2020) Convertino and Sather (2000)*
HR	Yes	↑	↑	Convertino et al. (2021)* Johnson et al. (2014)* Hinojosa-Laborde et al. (2014) Rea et al. (1991)* van Helmond et al. (2015)* van Helmond et al. (2016)*	Yes	Convertino et al. (2021)* Schiller et al. (2017)* Koons et al. (2020) Xiang et al. (2018)* Convertino and Sather (2000)*
PVR	Yes	↑	↑	Convertino et al. (2021)* Johnson et al. (2014)* Hinojosa-Laborde et al. (2014) van Helmond et al. (2015)* van Helmond et al. (2016)*	Yes	Convertino et al. (2021)* Schiller et al. (2017)* Xiang et al. (2018)* Convertino and Sather (2000)*
Respiration	Yes	↑	↑	Convertino et al. (2021)* Convertino et al. (2009)*	No	Convertino et al. (2013)*
EtCO_2_	Yes	↓	↓	Convertino et al. (2021)*	No	Convertino et al. (2013)*
SaO_2_ (SpO_2_)	Yes	↔	↔	Convertino et al. (2013) Johnson et al. (2014)* Hinojosa-Laborde et al. (2014) Koons et al. (2020)* Convertino et al. (2009)* Convertino et al. (2015)*	No	Convertino et al. (2013)* Koons et al. (2022)*
Hct	No	↓	↑	Johnson et al. (2014)* Hinojosa-Laborde et al. (2014) van Helmond et al. (2015)* van Helmond et al. (2016)*	Unk	-
Hgb	No	↓	↑	Johnson et al. (2014)* Hinojosa-Laborde et al. (2014) Koons et al. (2022)* van Helmond et al. (2016)*	No	Koons et al. (2022)*
ScvO_2_	No	↓	↔	Hinojosa-Laborde et al. (2014)	Unk	-
Lactate	Yes	↔	↔	Johnson et al. (2014)* Koons et al. (2020) Koons et al. (2022)*	No	Koons et al. (2020) Koons et al. (2022)*
Base excess	Yes	↔	↔	Johnson et al. (2014)*	Unk	-
HCO^3-^	Yes	↔	↔	Johnson et al. (2014)*	Unk	-
pH	Yes	↔	↔	Johnson et al. (2014)* Hinojosa-Laborde et al. (2014) Koons et al. (2020) Koons et al. (2022)* Convertino et al. (2009)*	No	Koons et al. (2020) Koons et al. (2022)*
WBC	Yes	↑	↑	van Helmond et al. (2015)*	Unk	-
PNA	Yes	↓	↓	Convertino et al. (2021)* Xiang et al. (2018)*	Yes	Convertino et al. (2021)* Schiller et al. (2017)* Xiang et al. (2018)*
SNA	Yes	↑	↑	Convertino et al. (2021)* Xiang et al. (2018)* Rea et al. (1991)*	Yes	Convertino et al. (2021)* Schiller et al. (2017)* Xiang et al. (2018)*
PRA	Yes	↑	↑	Hinojosa-Laborde et al. (2014)	Yes	Schiller et al. (2017)* Convertino and Sather (2000)*
VP	Yes	↑	↑	Johnson et al. (2014)* Hinojosa-Laborde et al. (2014)	Yes	Schiller et al. (2017)* Convertino and Sather (2000)*
NE	Yes	↑	↑	Hinojosa-Laborde et al. (2014) van Helmond et al. (2015)* van Helmond et al. (2016)*	Yes	Schiller et al. (2017)* Convertino and Sather (2000)*
Epi	Yes	↑	↑	Hinojosa-Laborde et al. (2014) van Helmond et al. (2015)* van Helmond et al. (2016)*	Unk	-
Coagulation	Yes	↑	↑	van Helmond et al. (2016)*	Unk	-
DO_2_	Yes	↓	↓	Convertino et al. (2021)* Koons et al. (2020) Koons et al. (2022)*	Yes	Koons et al. (2022)
CRM	Yes	↓	↓	Convertino et al. (2016)* Convertino et al. (2013)* Koons et al. (2020) Koons et al. (2022)* Convertino et al. (2015)*	Yes	Convertino et al. (2021)* Convertino et al. (2013)* Koons et al. (2020) Koons et al. (2022)*

HEM, hemorrhage; LBNP, lower body negative pressure; HT, high tolerant; LT, low tolerant; ABP, arterial blood pressure; CVP, central venous pressure; PP, pulse pressure; CO, cardiac output; SV, stroke volume; HR, heart rate; PVR, peripheral vascular resistance; EtCO_2_, respiratory end tidal carbon dioxide; SaO_2_, oxygen saturation of arterial blood; Hct, hematocrit; Hgb, hemoglobin; ScvO_2_, central mixed venous oxygen saturation; HCO^3-^, blood bicarbonate; pH, acidity; WBC, white blood cells; PNA, parasympathetic nervous activity; SNA, sympathetic nervous activity; PRA, plasma renin activity; VP, vasopressin; NE, norepinephrine; Epi, epinephrine; DO_2_, delivery of oxygen; CRM, compensatory reserve measurement. ↑, increase; ↓, decrease; ↔, no change. Refs 1, references supporting the changes resulting from central hypovolemia (HEM vs. LBNP); Refs 2, references with data that support the capability to distinguish HT from LT subjects; References with asterisks; data obtained from human subjects.

There are several general conclusions that can be made from the comparisons presented in Table 1. First, the similarities in magnitude and direction of changes in all but three physiological responses support the notion that LBNP represents a valid model for the study of hemorrhage physiology, and that sensors designed to measure hematocrit, hemoglobin or central mixed venous oxygen saturation of the blood should not be used for monitoring the hypovolemic state of hemorrhage. Second, the similarities in response to LBNP and blood loss have been verified in 21 of 24 (88%) physiological responses in humans (Johnson et al., 2014), with substantiation demonstrated in non-human primates (Hinojosa-Laborde et al., 2014) who display genetic similarities to humans (Convertino et al., 2021). Third, the majority of physiological responses to central hypovolemia (Table 1, second column from the right) induced by LBNP and hemorrhage cannot be used to develop precision medical algorithms because of failure to distinguish individuals with high tolerance to actual or simulated central hypovolemia (i.e., ‘good’ compensators) from those with low tolerance (i.e., at greatest risk for the early onset of hemodynamic instability or circulatory shock) (Convertino et al., 2023; Convertino et al., 2021; Cooke et al., 2004; Convertino et al., 2016; Schiller et al., 2017; Koons et al., 2022; Xiang et al., 2018; Convertino et al., 2000; Convertino and Sather, 2000; Convertino et al., 2015).

## 5 Quantitative overview of LBNP for simulation of hemorrhage

### 5.1 Hemodynamic responses to hemorrhage and LBNP in humans

**FIGURE 2:**
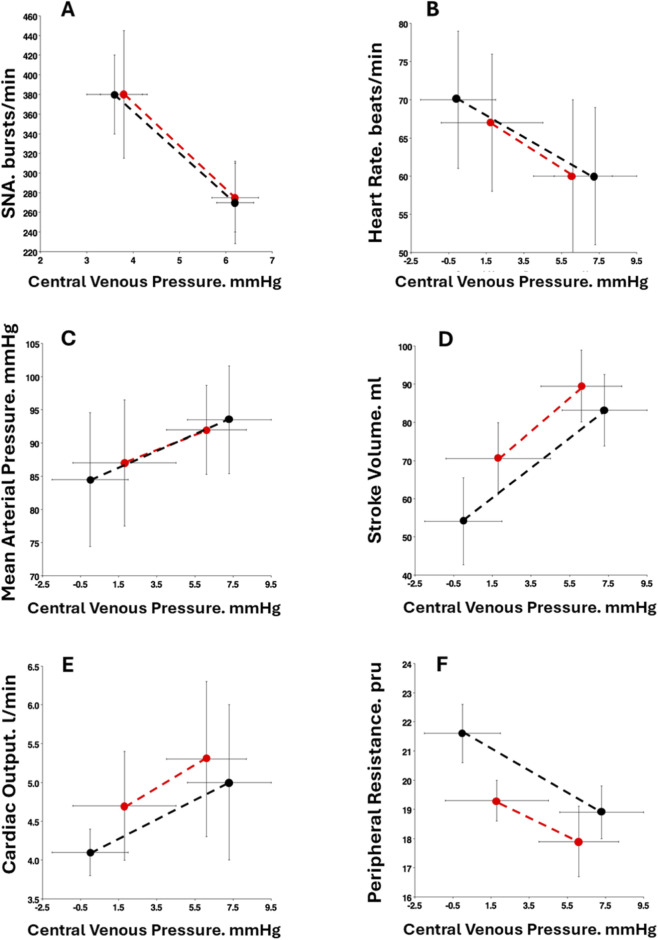
Sympathetic nerve activity (SNA; Panel **(A)**, heart rate (Panel **B**), mean arterial pressure (Panel **C**), stroke volume (Panel **D**), cardiac output (Panel **E**) and systemic peripheral resistance (Panel **F**) plotted against central venous pressure at baseline and immediately after protocol termination with Hemorrhage (red circles) and LBNP (black circles). Values are mean ±1 standard deviation. None of the stimulus-response slopes were statistically different between Hemorrhage and LBNP.

Demonstration that LBNP is a model of hemorrhage requires experimental designs in which various hemodynamic responses are quantitatively measured and compared during separate exposures to LBNP and bleeding in the same individuals. Lowered central venous pressure (CVP) associated with hypovolemia leads to reduced venous return, cardiac filling, stroke volume, cardiac output and hypotension despite compensatory elevations in heart rate and arterial vasoconstriction (Hinojosa-Laborde et al., 2014; van Helmond et al., 2015; van Helmond et al., 2016). In addition to comparisons of absolute values, central hypovolemia caused by LBNP exposure can also be used to calculate regression line slopes of hemodynamic responses per ΔCVP. Such slopes represent the stimulus-response reflex relationships that are responsible for hemodynamic control dictated by CVP as a physiological stimulus. The first documented study using a matched design approach was reported in 9 human subjects exposed to a non-hypotensive level of hemorrhage and one of LBNP (Rea et al., 1991). Sympathetic nerve activity (SNA; microneurography of the peroneal nerve) during hemorrhage compared to LBNP was obtained during this initial experiment (Cooke et al., 2004; Rea et al., 1991). When average elevations in sympathetic nerve activity (SNA) responded to reduced CVP with 450 mL blood withdrawal (Δ = −2.4 mmHg) and 10 mmHg LBNP (Δ = −2.7 mmHg) were compared (Cooke et al., 2004), the ΔSNA/ΔCVP stimulus-response slopes were essentially the same (Table 2; Figure 2, Panel A). The notion that LBNP provides a valid model to study hemodynamic responses to hemorrhage was advanced by data obtained during a subsequent seminal investigation (van Helmond et al., 2015) conducted at the Mayo Clinic where similar slopes were generated in hemodynamic parameters by similar reductions in CVP in 8 humans exposed to 1,000 mL blood volume withdrawal and 45 mmHg LBNP (ref. 8; Table 2; Figure 2, Panels B through F). Hemodynamic measurements during hemorrhage and LBNP exposure included heart rate (HR; continuous electrocardiogram recording), mean arterial pressure (MAP; arterial catheter), stroke volume (Model flow analysis for beat-by-beat area under the arterial blood pressure waveform), cardiac output (CO; product of stroke volume and heart rate), and total peripheral vascular resistance (TPR; calculated as MAP divided by CO). The order of the protocols was randomized; therefore, matching of CVP values between LBNP and BL was not possible due to subject safety. Despite the inability to match baseline stroke volume, cardiac output and peripheral resistance, stimulus-response relationships (i.e., slopes) for ΔSV, ΔCO and ΔTPR per ΔCVP remained equal (Figure 2, Panels D, E, and F). Thus, dynamic reflex responses to equal ΔCVP are similar between LBNP and hemorrhage.

**TABLE 2:** Average values of individual regression line slopes of hemodynamic responses per change in central venous pressure (Δ CVP) in humans (N = 8) exposed to 1,000 mL blood withdrawal (HEM) and 45 mmHg LBNP.

Hemodynamic responses	Hemorrhage	LBNP	P Value HEM slope vs. LBNP slope
Δ SNA/Δ CVP, % bursts/mmHg	−44	−43	>0.50*
Δ heart rate/Δ CVP, bpm/mmHg	−2.42	−1.53	0.158 †
Δ MAP/Δ CVP, mmHg/mmHg	1.12	1.20	0.237 †
Δ stroke volume/Δ CVP, mL/mmHg	4.22	3.88	0.636 †
Δ cardiac output/Δ CVP, mL/mmHg	0.10	0.10	0.642 †
Δ TPR/Δ CVP, pru x 10^9^/mmHg	−0.33	−0.36	0.524 †
Δ SaO_2_/Δ CVP, %/mmHg	−0.04	−0.04	0.999 †

* Data extracted from Cooke et al. (Cooke et al., 2004). † Data extracted from Johnson et al. (Johnson et al., 2014). CVP, central venous pressure; SNA, sympathetic nerve activity; MAP, mean arterial pressure; TPR, total peripheral resistance [pru in mmHg x (liters/min)^−1^; SaO_2_, oxygen saturation of arterial blood. Hemorrhage and LBNP, values are average slopes calculated across each subject; p-values represent t-test results across slopes indicating that the distributions of slopes are statistically similar.

### 5.2 White blood cell responses to hemorrhage and LBNP in humans

**FIGURE 3:**
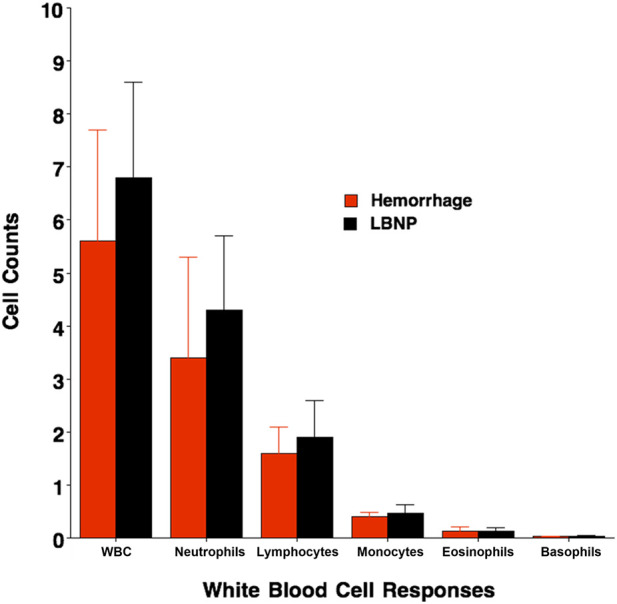
Values for white blood cell concentrations (10^9^ L^-1^) to include total white blood cells (WBC), neutrophils, lymphocytes, monocytes, eosinophils and basophils immediately after protocol termination with Hemorrhage (red) and LBNP (black). Values are mean (bars) ± 1 Standard Deviation (lines). None of the responses were statistically different between Hemorrhage and LBNP.

Reductions in central blood volume can represent a stimulus for an increase in white blood cells as an indication of immune system activation. Comparisons in stimulus-response slopes were calculated from average changes (Δ) from baseline rest in seven white blood cell populations per unit change in central venous pressure (Δ CVP) in 8 healthy human subjects. These immune system responses are summarized in Table 3. On one occasion, the volunteers were exposed to central hypovolemia by 1,000 mL of blood withdrawal (i.e., controlled hemorrhage) followed by a second session of central hypovolemia induced by exposure to 45 mmHg LBNP (van Helmond et al., 2015). All white blood cell responses increased with reductions in CVP caused by central hypovolemia, but none of the response slopes (Table 3) or absolute levels at protocol termination (Figure 3) were statistically indistinguishable (all p values ≥0.15) between LBNP and hemorrhage protocols.

**TABLE 3:** Average changes (Δ) in white blood cell responses per unit change in central venous pressure (Δ CVP) in humans during controlled hemorrhage and 45 mmHg LBNP.

Physiological response	Hemorrhage	LBNP
Δ total white blood cells/Δ CVP, (x10^9^ L^-1^)/mmHg	−0.09	−0.16
Δ neutrophils/Δ CVP, (x10^9^ L^-1^)/mmHg	−0.07	−0.09
Δ lymphocytes/Δ CVP, (x10^9^ L^-1^)/mmHg	−0.02	−0.07
Δ monocytes/Δ CVP, (x10^9^ L^-1^)/mmHg	0.01	−0.01
Δ eosinophils/Δ CVP, (x10^9^ L^-1^)/mmHg	0.000	0.001
Δ basophils/Δ CVP, (x10^9^ L^-1^)/mmHg	−0.006	−0.004

Data extracted from van Helmond et al. (van Helmond et al., 2015). CVP, central venous pressure.

### 5.3 Metabolic responses to hemorrhage and LBNP

Changes (Δ) in measurements of metabolic markers during the compensatory period of hemorrhage and LBNP are presented in Table 4 and Figure 4. Oxygen requirements to meet cellular energy demands exceed a reduction in the delivery of oxygen (DO_2_) that occurs secondary to diminished cardiac output (see section on hemodynamic responses to hemorrhage and LBNP) and oxygen-carrying capacity because of the reduced circulating red blood cells. A mismatch between tissue oxygen requirement and DO_2_ results in an oxygen deficit that requires increased extraction of blood oxygen stores and lowered partial pressures of blood oxygen. This ‘borrowing’ of oxygen reserves is an important mechanism during the ‘compensatory’ phase of hemorrhage but requires a greater proportion of the oxygen extracted from the blood by the tissue, leading to a progressive increase in oxygen extraction ratio (Convertino et al., 2021). As a result of increased oxygen extraction and buffering by blood bicarbonate, the acidity of the blood (i.e., blood pH) and blood lactate are not altered. Blood glucose is also increased during the compensatory phase of hemorrhage (Hinojosa-Laborde et al., 2014). Importantly, the responses of these metabolic markers are not statistically different between hemorrhage and LBNP (Table 4; Figure 4).

**TABLE 4:** Average metabolic responses (Δ) during exposure to similar levels of controlled hemorrhage and LBNP.

Physiological response	Hemorrhage	LBNP
Δ DO_2_, mL/min#	−110*	−139*
Δ blood pO_2_, mmHg#	−1.3	−1.7
Δ blood pCO_2_, mmHg #	−0.5	−0.4
Δ blood pH, acidity units#	0.00	−0.01
Δ blood lactate, mmol/liter †	0.10	0.05
Δ blood HCO_3_-, mmol/liter#	−0.4*	−0.4*

# Data extracted from Johnson et al. (Johnson et al., 2014). † Data extracted from Hinojosa-Laborde et al. (Hinojosa-Laborde et al., 2014). DO_2_, delivery of oxygen. *P < 0.05 vs. baseline rest.

**FIGURE 4:**
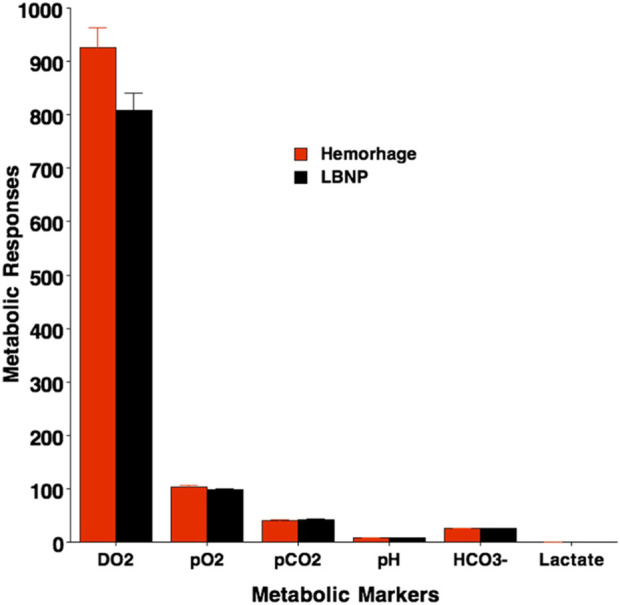
Metabolic responses immediately after protocol termination with Hemorrhage and LBNP. Delivery of oxygen (DO_2_, partial pressures of oxygen (pO_2_) and carbon dioxide (pCO_2_), blood acidity (pH), blood lactate, and blood bicarbonate (HCO_3_-) are presented. Values are mean (bars) ± 1 standard deviation (lines). No statistical differences were reported between Hemorrhage (red) and LBNP (black) values.

### 5.4 Coagulation responses to hemorrhage and LBNP in humans

**FIGURE 5:**
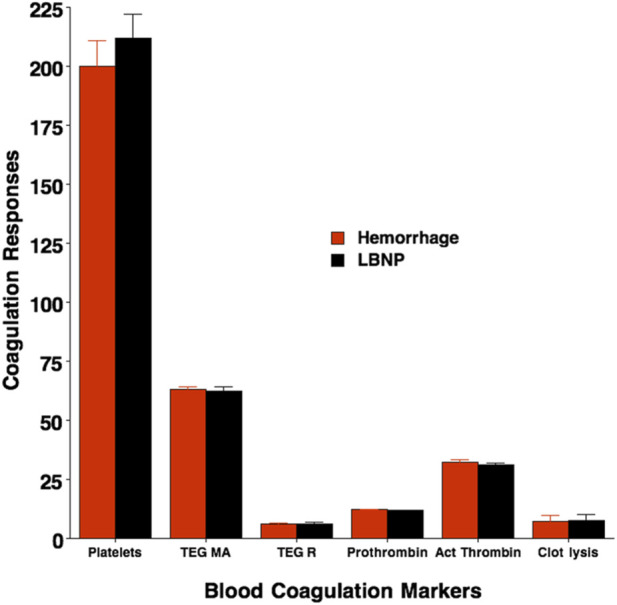
Coagulation responses immediately after protocol termination with Hemorrhage and LBNP. Platelet count (x10^9^ L^-1^), prothrombin time (seconds), activated partial prothrombin time (seconds), and thromboelastographic (TEG) measures of clot lysis at 60 min (%), time for initiation of clot formation (TEG R) and maximum amplitude (TEG MA) (platelet function/clot strength). Values are mean (bars) ± 1 standard deviation (lines). No statistical differences were reported between Hemorrhage (red) and LBNP (black) values.

Average responses in markers of coagulation system activation during the compensatory phase of hemorrhage and LBNP were measured in the healthy human volunteers who participated in the Mayo Clinic study before and after exposure to 1 L of blood withdrawal compared against 45 mmHg LBNP. Arterial blood samples were withdrawn before (baseline) and immediately after hemorrhage and LBNP. Prothrombin time and activated partial thrombin time were analyzed with standard coagulometric methods while clot formation, strength, and breakdown as assessed by thromboelastographic (TEG) measures. The results for coagulation responses are presented in Table 5; Figure 5 [reference 15]. There was little change in the time required for clot formation or the ability to break down clots as indicated by no statistical changes in prothrombin time, activated partial prothrombin time, and clot lysis before compared to after exposure of blood loss or LBNP. However, central hypovolemia induced by both blood withdrawal and LBNP resulted in increased clotting status as indicated by an elevated platelet count, and TEG measures of clot lysis at 60 min (%), reduced lower TEG R, and increased higher maximum amplitude (TEG MA). The average stimulus-response trajectories (slopes) measured per unit change in central venous pressure (ΔCVP) and TEG MA reflected a reduced time for the coagulation cascade to be initiated (TEG R), and increased clot strength, respectively. The changes in platelet count, TEG R and TEG MA were associated with significant increases in blood norepinephrine and epinephrine in both LBNP and hemorrhage protocols, suggesting a catecholamine-induced hypercoagulable state (van Helmond et al., 2016). Importantly, there were no statistically distinguishable differences (all p values ≥0.219) between LBNP and hemorrhage protocols for these coagulopathic markers.

**TABLE 5:** Average responses in coagulation markers in humans exposed to central hypovolemia induced by hemorrhage and LBNP. None of the responses was statistically different between Hemorrhage and LBNP protocols.

Physiological response	Hemorrhage	LBNP
Δ platelet count x10^9^/L	14 *	18 *
Δ prothrombin time, sec	0.1	−0.2
Δ activated partial prothrombin time, sec	−0.2	−1.2
Δ clot lysis @ 60 min, %	1.3	1.9
Δ TEG R/Δ CVP, min/mmHg	0.58 *	0.53 *
Δ TEG MA/Δ CVP, min/mmHg	−0.74 *	−0.63 *

Data were extracted from van Helmond et al. (van Helmond et al., 2016). CVP, central venous pressure; TEG, thromboelastography; R, reaction time; MA, maximum amplitude. *P < 0.05 from baseline values.

### 5.5 Summary of evidence

In summary, comparisons of hemodynamic, immune system, metabolic and neuroendocrine responses obtained from experiments in which human subjects have been exposed to both hemorrhage and LBNP using matched levels of CVP have, with few exceptions, repeatedly demonstrated that LBNP accurately mimics the overall physiological responses to actual blood loss during the compensatory phase of progressive central hypovolemia.

## 6 Safety of LBNP compared with blood withdrawal

LBNP provides a capability to test new monitoring technologies and wearable sensors without the confounding factors of disease or injury. Specific safety benefits of LBNP compared to blood withdrawal are outlined in Table 6.

**TABLE 6:** Benefits of LBNP compared to blood withdrawal trials.

#	LBNP	Controlled blood withdrawal	Benefit from LBNP
1	Lower safety risk to a human subject exists because of rapid reversal of central hypovolemia	There exists a higher risk of vasovagal syncope, infection due to venipuncture, and discomfort to human subjects	Although removal of blood can be stopped and reinfused into a subject, the time required to reintroduce blood can exacerbate risks of prolonged blood pressure instability can be significantly delayed during the decompensatory phase of shock
2	An LBNP protocol can be conducted with either stepwise or continuous profiles	Blood withdrawal usually requires a stepwise protocol	Blood withdrawal to the point of unexpected hemodynamic decompensation is difficult to execute safely without adversely compromising the subject’s health. In this respect, LBNP mimics hemorrhage conditions more safely than blood withdrawal
3	An LBNP protocol can be immediately reversed in both continuous and stepwise profiles with instant restoration of the chamber pressure	Reintroducing blood to a subject takes time and cannot quickly resolve a hemodynamic instability that has developed from the onset of decompensated shock	Hemodynamic instability can be especially critical for individuals who are decompensating since the delay in reintroducing withdrawn blood could compromise their safe recovery
4	A capability can be established to identify the physiology of individuals with high and low tolerance to central hypovolemia	Blood withdrawal cannot be designed to induce decompensation without significant compromise to the safety and wellbeing of the individual	Identifying individuals with low tolerance to hypovolemia allows for the testing of technologies that can identify patients at greatest risk for early onset of shock (i.e., development of precision medicine technologies)
5	A capability can be established to define the time course of compensation from baseline rest to hemodynamic instability	Blood withdrawal cannot be designed to induce decompensation without significant compromise to the safety and wellbeing of the individual	A capability to assess new technologies for early and accurate diagnostics of patient status cannot succeed without understanding the entire time course of compensation

## 7 Discussion and perspectives

The emergence of advanced innovative medical monitoring systems designed to provide early, and accurate assessment of life-threatening blood loss requires a novel approach for test and evaluation in humans. The use of human subjects requires that a safe testing model be used for monitoring system evaluations. The comparisons to actual blood withdrawal presented in this review paper provide verification that LBNP can be used to test the efficacy of new software algorithms and wearable sensors during varying levels of blood loss in humans.

The application of LBNP represents a new critical standard practice in the medical research community as a support function for design, testing and development of medical devices and wearable sensors. Such an approach would ensure for the first time the de-risking (i.e., evaluation of safety and performance) of various medical technologies. The ability to non-invasively reduce central blood volume that mimics the physiology of hemorrhage in healthy humans would provide a powerful tool in biomedical research, augmenting experimental research and technology development through detailed mechanistic and systematic investigations which are not safely available in experiments with other means such as actual blood withdrawal. Thus, human experiments using LBNP to simulate the physiology of hemorrhage are now emerging as an important tool for the test and development of novel machine learning algorithms, medical monitors, and wearable sensors (Convertino et al., 2023; Goswami et al., 2019; Cooke et al., 2004; Convertino et al., 2016; Schiller et al., 2017; Convertino et al., 2013).

Biomedical investigators have played a critical role in enabling the use of LBNP with experimental designs and electronic data collection that support the development of advanced medical monitoring technologies. In this regard, one of the most commonly accepted approaches for establishment of LBNP as a human hemorrhage model is to apply the FDA-recognized Standard for Assessing Credibility of Computational Modeling through Verification and Validation (ASME standard VandV 40) of Medical Devices specifically to LBNP. While this standard is meant to validate computational models, we have demonstrated how its principles can be applied to experimental platforms such as LBNP (Convertino et al., 2023).

LBNP provides consistent physiological stress for the study of progressive central hypovolemia (i.e., reduced central blood volume) in humans that allows for performance testing of medical devices and wearable sensors designed for early detection of hemorrhage. Specifically, LBNP can be used as a platform for testing any monitoring or sensor technologies that provide the capability to measure the physiological parameters listed in Table 1 because LBNP has been shown to accurately mimic these physiological responses when compared directly to actual blood loss (Johnson et al., 2014; Hinojosa-Laborde et al., 2014; Rea et al., 1991; van Helmond et al., 2015; van Helmond et al., 2016). LBNP provides an ability to test the safety or efficacy of a given diagnostic or therapeutic device in achieving its intended clinical effect. Such assessments can be used to inform the need for earlier lifesaving interventions and improve patient outcomes as well as provide the military and civilian emergency medical communities with the capability of evaluating the head-to-head performance assessments of various proposed monitoring and sensor devices.

LBNP has proven to be a valuable approach in early detection of hemorrhage, offering several notable benefits in both research and clinical settings. Based on the data presented in this review paper, LBNP has been shown to provide a safe and standardized way to simulate the physiological effects of hemorrhage without the need for creating actual blood loss in humans. This is particularly advantageous in device development, as it allows researchers to study physiological responses to hemorrhage in a laboratory-controlled environment, which enables accurate validity testing of wearable sensors. The capability to adjust the magnitude and transient profile of negative pressure values provides a novel approach to testing different levels of ongoing or controlled hemorrhage which would prove challenging in standard device trials.

LBNP offers a safe and non-invasive means of assessing an individual’s physiological response to hemorrhage. By subjecting humans to specifically controlled negative pressure profiles, clinicians and researchers can observe how the body’s compensatory mechanisms such as elevated heart rate and peripheral vasoconstriction respond to reduced central blood volume similar to that associated with actual hemorrhage (Convertino et al., 2021; Goswami et al., 2019). This insight is particularly valuable for early detection of ongoing blood loss, subtle clinical changes that may not be immediately apparent through traditional clinical assessments that depend on standard or traditional vital signs identified in the early stages of hemorrhage.

LBNP can also serve as a training tool for healthcare professionals. Medical personnel can gain experience in recognizing and managing hemorrhage-related conditions, improving their ability to respond effectively in real-life situations. By allowing for repeated practice in a controlled environment, LBNP can contribute to enhancing the skills and confidence of medical practitioners, ultimately leading to better patient outcomes in cases of hemorrhage and other clinical conditions of severe hypovolemia.

## 8 Limitations

Although LBNP is considered by institutional reviews to be greater-than-minimal risk, we are unaware of any published reports of compromise to health, safety or wellbeing in human subjects (Convertino et al., 2021). However, the first known reported results to quantify the safety of LBNP consisting of 187 human subjects (109 males, 78 females) revealed zero adverse events, including 50, 14 and 3 subjects exposed to 80, 90, and 100 mmHg LBNP, respectively (Convertino et al., 2025). Given that the assessment of LBNP presented in this review article has been historically conducted in healthy non-injured humans, we cannot dismiss that physiological responses outlined in Table 1 might be impacted by the complexity of trauma injury, and consequently the generalizability of LBNP as a human hemorrhage model. However, data collected from trauma patients suggest that there appears to be negligible impact(s) of trauma injury on algorithmic or sensor technologies generated from the use of LBNP (Convertino and Cardin, 2022).

## 9 Conclusion

In this review paper, we compared various hemodynamic, metabolic, hematologic, neuroendocrine, and coagulation responses during progressive central hypovolemia induced by LBNP and controlled hemorrhage (i.e., blood withdrawal). The result of our review provides compelling evidence to the clinical community that LBNP represents a non-invasive, minimal risk platform for testing advanced monitoring devices and wearable sensors for real-time hemorrhage.

## References

[B1] Convertino V. A. Cardin S. (2022). Advanced medical monitoring for the battlefield: a review on clinical applicability of compensatory reserve measurements for early and accurate hemorrhage detection. J. Trauma Acute Care Surg. 93, S147–S154. 10.1097/TA.0000000000003595 35271546

[B2] Convertino V. A. Sather T. M. (2000). Effects of cholinergic and β-adrenergic blockade on orthostatic tolerance in healthy subjects. Clin. Auton. Res. 10, 327–336. 10.1007/BF02322256 11324988

[B3] Convertino V. A. Sather T. M. (2000). Vasoactive neuroendocrine responses associated with tolerance to lower body negative pressure in humans. Clin. Physiol. 20, 177–184. 10.1046/j.1365-2281.2000.00244.x 10792410

[B4] Convertino V. A. Rickards C. A. Lurie K. G. Ryan K. L. (2009). Hyperventilation in response to reduction in central blood volume to near syncope. Aviat. Space Environ. Med. 80, 1012–1017. 10.3357/asem.2598.2009 20027847

[B5] Convertino V. A. Grudic G. Z. Mulligan J. Moulton S. (2013). Estimation of individual-specific progression to cardiovascular instability using arterial waveforms. J. Appl. Physiol. 115, 1196–1202. 10.1152/japplphysiol.00668.2013 23928113

[B6] Convertino V. A. Howard J. T. Hinojosa-Laborde C. Cardin S. Batchelder P. Grudic G. Z. (2015). Individual-specific, beat-to-beat trending of significant human blood loss: the compensatory reserve. Shock 44 (Suppl. 1), 27–32. 10.1097/SHK.0000000000000323 25565640

[B7] Convertino V. A. Wirt M. D. Glenn J. F. Lein B. C. (2016). The compensatory reserve for early and accurate prediction of hemodynamic compromise: a review of the underlying physiology. Shock 45, 580–590. 10.1097/SHK.0000000000000559 26950588

[B8] Convertino V. A. Koons N. J. Suresh M. (2021). Physiology of human hemorrhage and compensation. Compr. Physiol. 11, 1531–1574. 10.1002/cphy.c200016 33577122

[B9] Convertino V. A. Snider E. J. Hernandez-Torres S. I. Collier J. P. Eaton S. K. Holmes I. I. I. D. R. (2023). Verification and validation of lower body negative pressure as a non-invasive bioengineering tool for testing technologies for monitoring human hemorrhage. Bioengineering 10, 1226. 10.3390/bioengineering10101226 37892956 PMC10604311

[B10] Convertino V. A. Koons N. J. O’Hern E. D. Aden J. K. Milne S. J. Cardin S. (2025). Advanced decision support for monitoring casualties with hemorrhage: evidence against sole reliance on standard vital signs. J. Trauma Acute Care Surg. 99, S20–S26. 10.1097/TA.0000000000004699 40768656

[B11] Cooke W. H. Ryan K. L. Convertino V. A. (2004). Lower body negative pressure as a model to study progression to acute hemorrhagic shock in humans. J. Appl. Physiol. 96, 1249–1261. 10.1152/japplphysiol.01155.2003 15016789

[B12] Goswami N. Blaber A. Hinghofer-Szalkay H. Convertino V. A. (2019). Lower body negative pressure: physiological effects, applications and implementations. Physiol. Rev. 99, 807–851. 10.1152/physrev.00006.2018 30540225

[B13] Hinojosa-Laborde C. Shade R. E. Muniz G. W. Bauer C. Goei K. A. Pidcoke H. F. (2014). Validation of lower body negative pressure as an experimental model of hemorrhage. J. Appl. Physiol. 116, 406–415. 10.1152/japplphysiol.00640.2013 24356525 PMC4073981

[B14] Johnson B. D. van Helmond N. Curry T. B. van Buskirk C. M. Convertino V. A. Joyner M. J. (2014). Reductions in central venous pressure by lower body negative pressure or blood loss elicit similar hemodynamic responses. J. Appl. Physiol. 117, 131–141. 10.1152/japplphysiol.00070.2014 24876357 PMC4459917

[B15] Koons N. J. Nguyen B. Suresh M. R. Hinojosa-Laborde C. Convertino V. A. (2020). Tracking DO_2_ with compensatory reserve during whole blood resuscitation following controlled hemorrhage in baboons. Shock 53, 327–334. 10.1097/SHK.0000000000001367 32045396

[B16] Koons N. J. Moses C. D. Thompson P. Strandenes G. Convertino V. A. (2022). Identifying critical DO_2_ with compensatory reserve during simulated hemorrhage in humans. Transfusion 62, S122–S129. 10.1111/trf.16958 35733031

[B17] Rea R. F. Hamden M. Clary M. P. Randels M. J. Dayton P. J. Strauss R. G. (1991). Comparison of muscle sympathetic responses to hemorrhage and lower body negative pressure in humans. J. Appl. Physiol. 70 (3), 1401–1405. 10.1152/jappl.1991.70.3.1401 2033009

[B18] Schiller A. M. Howard J. T. Convertino V. A. (2017). The physiology of blood loss and shock: new insights from a human model of hemorrhage. Exp. Biol. Med. 242, 874–883. 10.1177/1535370217694099 28346013 PMC5407541

[B19] van Helmond N. Johnson B. D. Curry T. B. Cap A. P. Convertino V. A. Joyner M. J. (2015). Coagulation changes during lower body negative pressure and blood loss in humans. Am. J. Physiol. 309, H1591–H1597. 10.1152/ajpheart.00435.2015 26371166

[B20] van Helmond N. Johnson B. D. Curry T. B. Cap A. P. Convertino V. A. Joyner M. J. (2016). White blood cell concentrations during lower body negative pressure and blood loss in humans. Exp. Physiol. 101, 1265–1275. 10.1113/EP085952 27520090

[B21] Xiang L. Hinojosa-Laborde C. Ryan K. L. Rickards C. A. Convertino V. A. (2018). Time course of compensatory physiological responses to central hypovolemia in high- and low-tolerant human subjects. Am. J. Physiol. 315, R408–R416. 10.1152/ajpregu.00361.2017 29668322

